# Sunday Observance

**Published:** 1910-11-19

**Authors:** 


### SUNDAY OBSERVANCE.

The various tracts issued by the Lord's Day Observance Society, an organisation which was founded in the Early Victorian time, should draw public attention very forcibly to the fact that we are not a tolerant nation in the finest sense of the word. For it is incredible that a tolerant and tolerating people can allow the existence on its statute-book of that specimen of crystallised narrowmindedness known to lawyers as 21 Geo. III. c. 49, which provides that "any house, etc., open for public amusement or debate on a Sunday, to which persons shall be admitted by payment of money, etc., shall be deemed a disorderly house." It is not incredible that such associations as the Lord's Day Observance Society exist. There are many people who think they are the special instruments designed by Providence to see that the world goes right—men and women who, as old Father Latimer in one of his finest sermons phrased it, " snarl themselves into a fright because of their faith." These are the folk who can never realise the force of Molière's pertinent query:—

> Les intérêts du ciel! pourquoi vous changez-vous?
> Pour punir le coupable a-t-il besoin de nous?

Faddists by faith and compellants by conviction, they pride themselves on their firmness, and are insensitive alike to ridicule and reason. The sarcasm that created the Bishop in " Gil Blas," Tartuffe, Pecksniff, and, best of all, Drystubble, the intolerant coffee merchant, has left them untouched, and no amount of argument can convince them that the best method of enforcing the holiness of a creed or vindicating the truth of a faith is practical example and forbearance. For these reasons we will not waste our space and our arguments in attempting to criticise what we hold to be the absurd and immoral pretentions set forth in the literature which emanates from the office of the Lord's Day Observance Society. We are only concerned here with the professional and scientific aspects of the question of Sunday observance, and we will leave the Society with the earnest hope that it may do as much for our charitable institutions as the National Sunday League and other organisations that weekly transgress the egregious crystallisation of puritanical intolerance we have already alluded to.

There is a great deal of nonsense written and spoken about a weekly day of rest. No one attempts to hold that it is necessary to give a railway engine the benefits of a bank-holiday every seven days; and the human organism is a machine vastly more adaptable to average strain than any mechanical contrivance turned out in an engineering workshop. The point is, as every doctor knows, that in order to keep healthy every organism requires at certain periods change from the daily routine; but to suppose that that change is simply to consist in doing nothing under the pious name of rest is absurd. It is possible, no doubt, to be overworked to such an extent six days a week that remaining in bed all day on Sunday is the best holiday; but we hardly gather that this is the end which Sabbatarians have in view. Short of prostration, which demands a bed, every man requires exercise and outdoor amusement on Sunday, or whatever day of the week is holiday. He must get his roots fast in the earth again on the one day he escapes from the desk. Outdoor games, as our forefathers in " the spacious times of great Elizabeth " were fully aware, serve a specially useful purpose in this direction. The Church, which since the days of Moses, the father of all public health movements, has always provided for the physical condition of her people, recognised this by making Sunday a *holiday*, a day of feasting, which has made the word " holy " one to conjure with in every schoolroom ever since. The silvery laugh for comedy, to use no grosser irony, falls delicately on those who seek to make Sunday, the earliest public day of recreation on record, the dreadful thing of unhealthy repression which it was in the days of Dickens and in Augustus Hare's childhood. It is the medical profession which, as the guardian of national vigour, should make it plain to all authorities and private persons that bad hygiene means bad morality, and that proposals to curtail outdoor amusements and harmless recreation are a danger to the public health. Luckily, however, the places in which they may be adopted are as scanty as the fanatics by whom they are supported.

